# T Lymphocyte-Mediated Liver Immunopathology of Schistosomiasis

**DOI:** 10.3389/fimmu.2020.00061

**Published:** 2020-02-18

**Authors:** Bing Zheng, Jianqiang Zhang, Hui Chen, Hao Nie, Heather Miller, Quan Gong, Chaohong Liu

**Affiliations:** ^1^Department of Immunology, School of Medicine, Yangtze University, Jingzhou, China; ^2^Clinical Molecular Immunology Center, School of Medicine, Yangtze University, Jingzhou, China; ^3^Department of Intracellular Pathogens, National Institute of Allergy and Infectious Diseases, Bethesda, MD, United States; ^4^Department of Pathogen Biology, School of Basic Medicine, Tongji Medical College, Huazhong University of Science & Technology, Wuhan, China

**Keywords:** T lymphocyte, schistosomiasis, immunopathology, liver fibrosis, soluble egg antigen

## Abstract

The parasitic worms, *Schistosoma mansoni* and *Schistosoma japonicum*, reside in the mesenteric veins, where they release eggs that induce a dramatic granulomatous response in the liver and intestines. Subsequently, infection may further develop into significant fibrosis and portal hypertension. Over the past several years, uncovering the mechanism of immunopathology in schistosomiasis has become a major research objective. It is known that T lymphocytes, especially CD4^+^ T cells, are essential for immune responses against *Schistosoma* species. However, obtaining a clear understanding of how T lymphocytes regulate the pathological process is proving to be a daunting challenge. To date, CD4^+^ T cell subsets have been classified into several distinct T helper (Th) phenotypes including Th1, Th2, Th17, T follicular helper cells (Tfh), Th9, and regulatory T cells (Tregs). In the case of schistosomiasis, the granulomatous inflammation and the chronic liver pathology are critically regulated by the Th1/Th2 responses. Animal studies suggest that there is a moderate Th1 response to parasite antigens during the acute stage, but then, egg-derived antigens induce a sustained and dominant Th2 response that mediates granuloma formation and liver fibrosis. In addition, the newly discovered Th17 cells also play a critical role in the hepatic immunopathology of schistosomiasis. Within the liver, Tregs are recruited to hepatic granulomas and exert an immunosuppressive role to limit the granulomatous inflammation and fibrosis. Moreover, recent studies have shown that Tfh and Th9 cells might also promote liver granulomas and fibrogenesis in the murine schistosomiasis. Thus, during infection, T-cell subsets undergo complicated cross-talk with antigen presenting cells that then defines their various roles in the local microenvironment for regulating the pathological progression of schistosomiasis. This current review summarizes a vast body of literature to elucidate the contribution of T lymphocytes and their associated cytokines in the immunopathology of schistosomiasis.

## Introduction

Human schistosomiasis is a zoonotic parasitic disease caused by schistosomes, which are digenetic trematodes. In nature, many species of *Schistosoma* exist, but the main human pathogens are *Schistosoma japonicum, Schistosoma mansoni*, and *Schistosoma haematobium*. There is an estimate of more than 230 million people in tropical countries around the world infected with *Schistosoma* species (1). Infection occurs when cercariae, the free-living larval form of schistosomes, are released from freshwater snails and penetrate a human host's skin, where they may remain in the host epidermis for ~72 h (2). Then, cercariae transform into schistosomula, the parasitic larvae form that enter the vasculature and travel via the pulmonary artery to the lungs, which they are then referred to as lung schistosomula (3). After exiting the lungs, schistosomula re-enter the venous circulation and finally migrate to the perivesicular venules (*S. haematobium*) or mesenteric venules (*S. japonicum, S. mansoni*), where they mature into worms and form copulating pairs. Paired adult worms migrate to the intestinal venous vasculature to release their eggs. Of the fertilized eggs released by the female, a portion is discharged via emunctory routes and hatch in freshwater, releasing free-living ciliated miracidia that invade snails and continue on the life cycle. However, a large portion of *S. haematobium* eggs may become trapped in the bladder and urogenital system, while the eggs of *S. japonicum* and *S. mansoni* become lodged in the intestinal wall and liver (4). The eggs then induce a local granulomas inflammatory response. The granulomas mainly consist of lymphocytes, macrophages, and eosinophils, which contain egg proteolytic enzymes to prevent tissue damage; however, the egg-induced granuloma also leads to chronic schistosomiasis. In this review, we will focus on *S. japonicum* and *S. mansoni*, both of which have been extensively studied and have also been used in mouse models to study liver immunopathology.

The basic pathological process of schistosomiasis includes acute and chronic phases of the disease. Acute schistosomiasis (sometimes referred to as Katayama fever) occurs most often in schistosome-endemic regions to naive individuals and to those who experience heavy reinfection. The major clinical manifestation of acute schistosomiasis includes a sudden onset of fever, fatigue, urticaria, eosinophilia, and abdominal tenderness. These symptoms occur weeks to months after schistosome infection because of worm maturation, egg production, and egg antigen-induced inflammatory granulomatous response (4, 5). Chronic schistosomiasis involves immune responses to the eggs within the liver and intestine, which causes formation of granuloma (6–9). Over time, the granulomatous response is gradually downregulated, leading to the progression of relatively tolerable chronic intestinal schistosomiasis. However, some patients will develop life-threatening hepatosplenic schistosomiasis accompanied by extensive hepatic and periportal fibrosis, portal hypertension, ascites, and gastrointestinal hemorrhage.

T lymphocytes are generally classified into CD4^+^ T helper (Th) cells and CD8^+^ cytotoxic T lymphocytes (CTLs). Th cells are important for host humoral and cellular immune responses against parasitic infections (6). The Th cells are further classified into several distinct Th phenotypes [Th1, Th2, Th17, follicular helper T cell (Tfh), Th9, and regulatory T cells (Tregs)] according to cytokine production and specialized functions. This review focuses on the role of various Th subsets and their associated cytokines in the immunopathogenesis of schistosomiasis.

## The Role of Th1/Th2 Responses in the Immunopathology of Schistosomiasis

An accumulation of evidence suggests that Th cells are involved in the immunopathogenesis of schistosomiasis. In athymic nude mice or mice without Th cells resulted in decreased granuloma size after infection by schistosomes (10, 11). The role of Th1/Th2 responses in schistosomiasis has been intensely investigated and reviewed (6, 9, 12). In the acute illness, parasite antigens elicit a moderate Th1 response, which is characterized by increased levels of proinflammatory cytokines, including tumor necrosis factor alpha (TNF-α), interferon gamma (IFN-γ), interleukin-1 (IL-1), and IL-6 (13). About 5–6 weeks post-infection, the schistosomula develop into mature worms. Female worms release fertilized eggs that stimulate the immune response via their soluble egg antigens (SEAs). SEA induces resident macrophages to secrete inflammatory cytokines and chemokines that stimulate the influx of lymphocytes, neutrophils, and monocytes, which initiates circumoval granulomatous inflammation (14). A clinical study comparing acutely and chronically infected patients showed that the level of TNF-α was elevated in the plasma of acutely infected patients and their peripheral blood mononuclear cells spontaneously secreted high levels of IL-1 and IL-6 and detectable levels of IFN-γ, while chronically infected patients produced little TNF-α or IFN-γ. Thus, higher levels of IFN-γ and proinflammatory cytokines in patients with acute illness show a dominant Th1 response (15). During the acute phase, the SEA-specifically activated CD4^+^ Th cells release the Th1-type cytokines, IL-2 and IFN-γ, which mediate the establishment of early granulomas. Immunocytochemical examination *in situ* have confirmed the presence of Th1-type cytokine-producing cells within local microenvironments of the lesion in early granuloma formation (16). Although the granulomatous response is detrimental to the liver because of subsequent progression of hepatic fibrosis, the egg-induced granuloma is beneficial to the host. If the eggs are not sequestered effectively, continuously secreted egg antigens act as a stimulus and lead to uncontrolled inflammatory responses and permanent tissue damage. For instance, IL-4-deficient mice that cannot mount a normal Th2 response develop an unchecked Th1 response and die earlier than immunity intact mice when infected by *S. mansoni* (6). Similarly, in a Tamoxifen-induced IL-4 receptor α (IL-4Rα)-deficient mouse model, interrupting IL-4Rα-mediated signaling during the acute stage decreased protective Th2 responses, leading to severe disease and premature death (17). Therefore, moderate Th1 responses are involved in the acute schistosomiasis and early granuloma formation, whereas excessive polarization toward the Th1 response is detrimental to the host. The Th1 response during the early stage of schistosomiasis is downregulated by IL-4 and IL-10. IL-10-/IL-4-deficient mice develop extremely polarized Th1-type cytokine IFN-γ responses that lead to 100% mortality during the acute illness (18).

The immune response and immunopathology of schistosomiasis are a consequence of CD4^+^ T-cell sensitization to egg antigens. Some of the major components of egg are implicated in the Th response in schistosomiasis, including glycoprotein IPSE/α1, ω1 (19, 20), lacto-*N*-fucopentaose III (21), and *S. mansoni*-p40 (Sm-p40) (22), of which Sm-p40 is the most abundant egg component that can induce a strong Th1-polarized response (23). IPSE/α1 induces a mixed Th1/Th2 response and promote the development of hepatic granuloma (19), while lacto-*N*-fucopentaose III and ω1 directly act on dendritic cells (DCs) to enhance the Th2 response (21, 24). In addition, Th1-type responses could also be induced by the schistosome vaccine candidates MAP4 (25), egg-derived r38 (22, 26), rSmLy6B, rSmTSP6, and rSmTSP7 (27), and rSjCRT (28).

Compared to the Th1 response, the Th2 response exerts anti-inflammatory effects and regulates Th1-mediated immunopathology. SEA is considered to be the key factor in driving the dramatic transition from a Th1- to a Th2-dominated response. During the Th response transition, the interaction of CD40–CD40L, CD80/86–CD28, and B7-related protein 1-inducible costimulator (ICOS) are required for egg antigen-induced Th2 responses (29–31). In correlation with the Th2 transition, the cytokine profile is changed in that IFN-γ production is decreased, whereas Th2-type cytokines IL-4, IL-5, and IL-13 are increased (32). This has been shown in lymphocytes isolated from liver granulomas at 8 weeks, which secrete IL-4 and IL-5, but not IFN-γ. The mechanism underlying this switch to Th2 response has been deeply investigated. Lymphocytes from *S. mansoni* ova-infected signal transducer and activator of transcription (STAT6)-deficient mice produce minimal levels of Th2-type cytokines and enhanced IFN-γ production, which greatly reduces the size of pulmonary and hepatic granulomas (33). IL-4 signal is critical for the development of the Th2 response and animals treated with anti-IL-4 showed decreased Th2-type IL-4, IL-5, and IL-10 and increased Th1-type IL-2 and IFN-γ (34). IL-4 is recognized as the dominant cytokine for granuloma development and injection with neutralizing antibodies against IL-4 significantly suppresses splenic cell proliferation and hepatic granulomatous inflammation (35). Nevertheless, other studies showed that infected IL-4 knockout (KO) mice still have the ability to generate egg granulomas. Surprisingly, infected IL-4Rα-deficient mice exhibit only minimal hepatic granulomas and fibrosis, even though Th2-type cytokine production is similar to infected IL-4 KO mice, which demonstrates that the IL-4R signaling pathway, rather than IL-4, may be essential for egg granulomas (36). However, another Th2-type cytokine, IL-13, is known to be involved in granulomatous inflammation and fibrosis through the IL-4Rα signaling. Blocking IL-13 has been shown to be highly effective in treating an established and ongoing *S. mansoni* infection-induced fibrosis (37, 38). These results were further confirmed by studies using schistosome-infected IL-13 and IL-4-/IL-13-deficient mice (39). Furthermore, IL-13-deficient mice exhibit increased survival time, demonstrating the important role of IL-13 in the pathogenesis of schistosomiasis. *In vitro* studies revealed that IL-13 stimulates collagen production in fibroblasts and has a direct role in collagen homeostasis of normal human skin and keloid fibroblasts (40, 41). Overall, it is known that IL-4 and IL-13 play redundant roles in the schistosomiasis granulomatous response, but IL-13 is not dependent on profibrotic cytokines or affected by Th1/Th2 cytokines.

The source of Th2-type cytokines are not only produced by Th2 cells but also secreted by other innate lymphocytes, such as type 2 innate lymphoid cells (ILC2s). The three epithelial cell-derived cytokines, IL-33, IL-25, and thymic stromal lymphopoietin (TSLP), act as crucial initiators of Th2 responses to induce ILC2s to produce IL-13, therefore promoting Th2-type immunity (42–44). It has been reported that IL-33 treatment promotes a Th2 response together with increased liver immunopathology, and these effects are prevented by anti-IL-33 monoclonal antibodies. Furthermore, IL-33 is a requisite for IL-13-, but not IL-4, driven Th2 responses during the pathology (45). Subsequent studies have further confirmed that IL-33 is involved in initiating Th2 pathology after schistosome infection via regulating IL-13 expression in hepatic stellate cells (46) and inducing polarization of M2 macrophages (47). However, another study demonstrated an overlapping role of IL-33, IL-25, and TSLP in a schistosome-induced lung granuloma and liver fibrosis model. They showed that simultaneous disruption of IL-33, IL-25, and TSLP signaling inhibited the progression of Th2 cytokine-driven liver fibrosis but that individual disruption of each had no effect (48).

In addition to SEA-induced specific Th2 responses, parasite-derived cysteine peptidases are also responsible for Th2 responses. For instance, during acute schistosomiasis, injection of outbred mice with *S. mansoni* cathepsin B1 (SmCB1) or *S. mansoni* cathepsin L3 (SmCL3) develops a polarized Th2-type immune environment that is harmful for the development of *S. mansoni* larvae and leads to significant reduction in worm burden and liver egg counts (49). A similar study also showed the effectiveness of a cysteine-peptidase-based vaccine that protects hamsters from schistosomiasis *haematobium* by inducing Th2 immune responses (50). Therefore, the cysteine-peptidase-based vaccine has shown great potential to be further used in non-human primates, and even in humans, through inducing protective Th2 immune responses.

Although a strong Th2 response seems detrimental to the host, a Th2 response is indispensable for the survival of the host. Th2-deficient IL-4^−/−^ mice are highly susceptible to infection and develop severe acute cachexia followed by death (51). Depletion of the Th2 response against the eggs results in tissue damage and increased host mortality and liver pathology due to proinflammatory Th1-type responses (52, 53). Clinical cases also demonstrate that more severe forms of hepatosplenic schistosomiasis is linked to low levels of Th2-type IL-5 and increased Th1-type IFN-γ and TNF-α (54). Therefore, Th2 immunity acts as a double-edged sword: on the one side, it protects the host to decrease the overall pathology and prevent excessive granulomatous inflammation, but on the other side, it causes liver immunopathological damage. Thus, the Th2-response-mediated egg granuloma is a necessary evil for host survival. Therefore, maintaining the proper balance of Th1/Th2 responses is important to control the excessive pathology of schistosomiasis. Previous studies demonstrated that excessively polarized Th1- or Th2-type cytokine responses induce different but equally detrimental pathologies after infection (18). IL-4/IL-10 double-deficient mice develop highly polarized Th1-type cytokine responses, exhibited by rapid weight loss during egg production and 100% acute mortality by week 9 post-infection. In contrast, IL-12/IL-10 double-deficient mice with highly polarized Th2-type cytokine responses develop increased hepatic fibrosis and mortality during the chronic stages of infection (18). Both Th1 and Th2 phases are downregulated by endogenous IL-10, which is produced by macrophages and T cells (55). Thus, IL-10 acts as an important regulator to prevent excessive Th1 and Th2 responses during the development of schistosomiasis.

## Th17/IL-17 Exacerbate the Egg-Induced Liver Immunopathology

The first notion of Th17 cells playing a role in schistosomiasis egg-induced granuloma formation came from experiments using knockout mice unable to produce IL-23, which drives the production of IL-17 by Th17 cells. In these experiments, IL-12p40^−/−^ mice, incapable of producing IL-12 or IL-23, were highly resistant to liver pathology, whereas IL-12p35^−/−^ mice, able to produce IL-23 but not IL-12, developed severe granuloma lesions (56). Granuloma formation is associated with high levels of IL-17 and treatment with anti-IL-17 neutralizing antibodies significantly inhibited hepatic granulomatous inflammation (56). Therefore, the IL-17-producing CD4^+^ T-cell population driven by IL-23 was recognized as a separate lineage and designated as Th17 cells that contribute to severe immunopathology in schistosomiasis (56, 57).

The IL-23–IL-1–IL-17 axis plays an essential role in the development of severe forms of schistosomiasis (58). A study conducted on mice lacking the IL-23-specific subunit p19 revealed an impaired liver immunopathology together with a marked decrease in IL-17 in the granulomas, but not in the draining mesenteric lymph nodes (59). Subsequent studies conducted on high pathology-prone CBA mice and low pathology-prone C57BL/6 mice identified IL-23 and IL-1, derived from DCs stimulated by live schistosome eggs, as the critical host factors that drive IL-17 production (60, 61). *S. mansoni*-infected CBA mice possess more IL-17-producing cells in the spleen and granulomas when compared with C57BL/6 mice (62). It was later determined that egg antigens, but not adult worm antigens, preferentially induce the generation of Th17 cells. Lowering IL-17 levels by neutralizing anti-IL-17 antibodies can increase the parasite-specific antibody levels and supply partial protection against *S. japonicum* infection in mice (63). In addition, anti-IL-17 antibody markedly inhibited hepatic granulomatous inflammation and hepatocyte necrosis partly through reducing the proinflammatory cytokines/chemokines and infiltrating neutrophils (64). Similar results were obtained with *S. japonicum-*infected IL-17RA-deficient mice that displayed decreased granulomatous inflammation, hepatic fibrosis, improved liver function, and high survival (65). Rutitzky et al. investigated the role of IL-17 and IFN-γ in schistosomiasis immunopathology using mice lacking either one or both cytokines. They found that IL-17-deficient mice show significantly reduced immunopathology associated with the increased levels of IFN-γ, whereas IFN-γ-deficient mice displayed exacerbated immunopathology as well as increased levels of IL-17. Hence, IL-17 plays a powerful pathogenic role in severe immunopathology in murine schistosomiasis that normally is restrained by IFN-γ (66). It has also been reported that T-bet^−/−^ mice have significantly increased egg-induced hepatic immunopathology, with the absence of IFN-γ and increased IL-23p19, IL-17, and TNF-α in granulomas. Thus, T-bet-dependent signaling negatively regulates Th17-mediated schistosomiasis immunopathology (67).

Recent reports found that ICOS is essential for pathogenic-induced Th17 cell development. This was discovered by Wang et al., who found that ICOSL KO mice had lower levels of Th17-associated cytokines (IL-17/IL-21), IL-13, and TGF-β1, which is correlated with improved survival rate, alleviated liver granulomatous inflammation, and hepatic fibrosis development (68). In addition, CD209a (C-type lectin receptor), expressed on DCs, also proved to be essential for the development of Th17 cell responses in murine schistosomiasis (61, 69). CD209a-deficient CBA mice had decreased Th17 responses and developed reduced egg-induced liver immunopathology (69, 70). Clinical studies also demonstrated the positive correlation between the percentage of Th17 cells and bladder pathology in *S. haematobium*-infected children (62).

The above findings demonstrate that Th17/IL-17 exacerbate the egg-induced liver immunopathology in schistosomiasis. However, it should be noted that a recent study found that acute schistosome infection induced a transient Th17 response to cathepsin B1 cysteine proteases secreted by the worms and that, this early, Th17 response may determine the pathogenic progression of the infection (71).

Although the role of Th17/IL-17 in schistosomiasis liver immunopathology has been defined, the source of IL-17 is not completely clear. Generally, IL-17-producing cells include Th17 cells, CTL cells, γδT cells, and natural killer T cells. Some researchers ignore the source of IL-17, and others found that Th17 cells are the major IL-17-producing cell population that contributes to pulmonary granuloma induced by *S. japonicum* (72). However, some studies showed that innate γδ T cells are the major IL-17-producing cells that contribute to the formation of granuloma in murine schistosomiasis (73, 74). The above discrepant findings may result from the different granuloma models. In addition, aforementioned ILC2s mediate Th2-type immunity through secreting IL-13. The ILC3s, as counterparts to Th17 cells, engage in Th17 immunity-mediated mucosal homeostasis and defense (75). However, the exact role of ILC3s has not been clarified yet in the pathology of schistosomiasis.

Thus far, numerous studies have promoted our understanding of the basic immunopathogenesis of schistosomiasis; however, it should be noted that schistosome-induced liver immunopathologies are associated with *Schistosoma* species and host. Examples of this include that the cellular composition of the *S. japonicum* egg-induced granulomas are mainly neutrophils, whereas *S. mansoni*-induced granulomas consist of a higher ratio of mononuclear cells and eosinophils, with lower numbers of neutrophils (76, 77). The differences between *S. japonicum*- and *S. mansoni*-induced hepatic granuloma could be attributed to the secreted specific leukocyte-associated chemokines at the site of inflammation (77). A host's genetic background also affects infection intensity and pathology of schistosomiasis (78, 79). Nevertheless, most researchers perform their studies using BALB/c and C57BL/6 strain mice. Actually, schistosome-infected BALB/c and C57BL/6 mice only develop mild hepatic granulomatous inflammation; however, CBA and C3H mice develop a severe pathology with larger size and poorly confined granulomas (80). As previously discussed, mouse strain-dependent schistosomiasis pathology may arise from the difference of Ag-specific Th responses (62), such as *S. mansoni*-infected CBA mice displaying exacerbated granulomatous lesions when compared to C57BL/6 mice because of high ratios of IL-17-producing cells in the granulomas (62).

## Tfh Promotes Liver Pathology of Schistosomiasis

Tfh cells are a specialized Th subset equipped to provide B cell help (81). Gene microarrays revealed that the transcriptional profile of Tfh cells is different from Th1, Th2, and Th17 cells (82). Tfh cells are mainly located in the periphery of B-cell follicles in secondary lymphoid organs and are identified by expression of various molecules, such as surface receptors CXCR5, programmed death 1 (PD-1), ICOS, the transcription factor B-cell lymphoma 6 (Bcl-6), and the cytokine IL-21 (83–85). Tfh cells were found to differentiate from Th2 cells in germinal centers responding to SEA (86). *S. japonicum* recombinant protein, SjGST-32, also has the ability to induce the formation of Tfh cells in BALB/c mice, which promotes humoral immune responses and long-lived memory B cells (87). Functional studies on Tfh cells showed that downregulation of their cellular development leads to immune deficiencies and that Tfh cells are closely associated with autoimmune and chronic inflammatory disease (85, 88). *S. mansoni* eggs induce differentiation of Tfh, which is highly dependent on Notch expression on T cells. Notch-deficient mice show impaired germinal center formation and decreased secretion of high-affinity antibodies (89). Tfh expansion and antibody production in response to schistosome infection are negatively regulated by B7-H1 (programmed death ligand 1) that is expressed on B cells (90). Immunization of B7-H1^−/−^ mice with SEA leads to increased numbers of Tfh cells compared to wild-type mice (90). Recent studies have demonstrated that Tfh cells promote liver granulomas and fibrogenesis in mice infected with *S. japonicum* (91, 92). Wang et al. found that, in murine schistosomiasis, Tfh cells accumulate in the splenic germinal center and that the Tfh phenotypic molecule, Bcl-6, and the Tfh-type cytokine, IL-21, correlate with progression of liver fibrosis (92). In addition, clinical studies have shown that Tfh cells are involved in immune responses for both acute and chronic human schistosomiasis (93, 94). The frequency of circulating PD-1^+^CXCR5^+^CD4^+^ Tfh cells in the peripheral blood is increased in both acute and chronic schistosomiasis patients relative to healthy controls. However, the difference between acute and chronic schistosomiasis is that there is no correlation between percentages of PD-1^+^CXCR5^+^CD4^+^ Tfh cells, memory B cells, or the level of immunoglobulin G (IgG) specific to *S. japonicum* antigen in acute schistosomiasis patients; however, frequency of PD-1^+^CXCR5^+^CD4^+^ Tfh cells is positively correlated to the levels of IL-21 in sera and the levels of SEA-specific antibody in chronic schistosomiasis patients (93, 94). These findings demonstrate that Tfh cells might exhibit distinct mechanisms to regulate the immune response between acute and chronic schistosomiasis. In addition, IL-4-producing Tfh cells are presumed to be important for naturally acquired resistance to schistosome reinfection (95).

## Th9 Cells and Egg-Induced Hepatic Granulomatous and Fibrosis

Th9 cells are another unique subset of CD4^+^ T cells recently characterized by their production of cytokine IL-9 after activation. The specific transcription factors for Th9 cells include PU.1 and IRF-4 (96, 97). Before discovery of the Th9 cell, IL-9 was thought to be a Th2-specific cytokine. Until 2008, it was reported that IL-9 could be secreted exclusively by distinct IL-9^+^IL-10^+^ Th cells lacking suppressive function (98). Th9 cells have been implicated in many diseases, such as allergic inflammation, autoimmune disorders, as well as helminth infections (96, 99, 100). Zhan et al. investigated the dynamics of splenic Th9 cells and IL-9 expression in liver and serum and found that the proportion of splenic Th9 cells and levels of IL-9 were significantly higher in *S. japonicum*-infected mice compared to uninfected controls. Moreover, dynamic changes of Th9, IL-9, and PU.1 levels were consistent with hepatic egg granulomatous inflammation (101). In agreement with these studies, Li et al. additionally showed that anti-IL-9 monoclonal antibody treatment significantly inhibits *S. japonicum*-induced hepatic granulomatous and fibrosing inflammation (102). The above findings indicate that Th9 cells may be involved in immunopathogenesis in murine schistosomiasis. However, a clinical investigation showed that the level of IL-9 in serum had no significant difference between acute and chronic schistosomiasis patients (103). Therefore, further studies are needed to clarify the defining roles of Th9 cells and IL-9 in the pathology of human schistosomiasis.

## Tregs Regulate the Granulomatous Inflammation and Fibrosis

In 1995, Sakaguchi et al. introduced regulatory T cells to the field of immunology (104). Tregs are a separate lineage of T cells that are responsible for maintaining immunological homeostasis, suppressing potentially deleterious activities of Th cells, as well as mediating the magnitude of immunity against invading pathogens (105). It has been conceded that two main types of Tregs exist: one is termed “inducible” Tregs (iTregs), which responds to infectious challenge, and the other is termed “natural” Treg (nTregs), which is an endogenous Tregs (106). The forkhead box protein 3 (Foxp3) is a unique transcription factor that can be used to separate nTregs from iTregs that have similar regulatory properties (107). The nTregs develop from a normal process of maturation in the thymus and serve as an essential subset of T cells in the periphery. The specific markers for nTregs include CD25, the T-cell inhibitory co-receptor CTLA-4, and the glucocorticoid-inducible TNF receptor (108, 109). The iTregs originate from conventional CD4^+^ T cells that are exposed to specific stimulatory factors, such as a cocktail of cytokines or drugs (110). Currently, the iTregs identified include IL-10-producing Tr1 cells, TGF-β-producing T helper type 3 (Th3) cells, and regulatory CD4^+^CD25^+^Foxp3^−^ cells (111, 112). Both iTregs and nTregs exert suppressive/regulatory effects to restrict immune-mediated pathology.

So far, numerous studies have demonstrated that both nTregs and iTregs represent key players in the regulation of schistosomiasis pathology. During schistosome infection, Tregs suppress DC activation, mediate Th2 responses, and inhibit granuloma development and fibrosis. After infection with *S. mansoni*, the percentage of granuloma nTregs (CD4^+^ CD25^+^ Foxp3^+^) has a significant increase at 8 and 16 weeks of the infection (113). Similarly, schistosome eggs show the ability to induce a significant Foxp3^+^ Treg cell response, which suggests that SEA may be the most potent inducer for the generation of nTregs during infection (114). Except for SEA, schistosome-derived molecules, such as lysophosphatidylserine (lyso-PS), identified as the TLR2-activating molecule, can also actively induce the development of IL-10-producing Tregs (115). Although the percentage of Tregs are elevated either following infection or egg immunization, the natural ratio between nTregs and effector T cells is remarkably stable during the progress of the egg-induced inflammation, suggesting that the expansion of effector T cells maybe closely regulated by the nTregs response.

With the progression into the chronic stage of egg-induced inflammation, the phenotype of nTregs is changed so that the frequency of CD103-expressing nTregs is strongly increased. CD103 binds integrin β7 to form the complete integrin molecule αEβ7, which is an activation marker for nTregs. Thus, increasing the CD103-expressing nTregs is required for immunosuppression during chronic schistosomiasis (116). Although numerous studies have characterized nTregs during schistosome infection, the exact mechanism of how nTregs regulate immunopathology is not yet clear. To address the functional role of Tregs in schistosomiasis, many studies used the CD4 and CD25 sorting of Tregs; however, this makes it difficult to distinguish the function of either Tregs because CD4^+^CD25^+^ compartment includes both nTregs and iTregs. Nevertheless, Baumgart et al. established nTregs depleted mice to evaluate the role of nTregs in egg-induced inflammation. They found that both IFN-γ and IL-4 responses were increased following immunization, demonstrating that nTregs suppress both Th1 and Th2 responses (116), which is likely not associated with IL-10 (114).

In addition to nTregs, schistosomiasis infection also induces the production of iTregs (117, 118). It has been reported that IL-10, secreted from iTregs and Th2 cells, inhibits IL-12 production by CD40 agonist-stimulated DC and iTregs, thus suppressing development of egg-specific Th1 responses during schistosomiasis (118). Furthermore, IL-10-secreting iTregs isolated from the granuloma of chronically infected mice can inhibit CD4^+^ T-cell proliferation (118), which is different from nTregs that do not appear to control the proliferation of T cells *in vivo* (116). Therefore, it is possible that both nTregs and iTregs have the ability to suppress both Th1 and Th2 responses. To investigate the importance of Tregs for controlling schistosomiasis liver pathology, adoptive transfer purified populations of CD25-depleted CD4^+^ T cells into RAG-deficient mice (lack of mature T or B lymphocytes) lead to increased weight loss, liver damage, and mortality following infection, suggesting a strong capacity for Tregs to suppress liver pathology (117).

## What About the Role of CD8^+^ T Cells?

The role and mechanism of CD8^+^ T cells in the process of schistosomiasis has been a somewhat neglected area of study. Early studies conducted in the pulmonary granuloma model demonstrated that CD8^+^ T-cell deficiency increased granuloma formation by 70%, which was attributed to CD8^+^ T-cell inhibition of Th2 maturation (119). However, a subsequent study found that granuloma formation and hepatic granulomatous reaction were unchanged in CD8^+^ T-cell-deficient mice (120). Another study, using major histocompatibility complex (MHC) class II or I mutant mice to examine the role of CD4^+^ and CD8^+^ T cells in the pathology of schistosomiasis, found that schistosome-infected MHC I mutant mice developed normal granulomatous lesions, while in contrast, MHC II mutant mice failed to form egg granuloma (121). Therefore, CD8^+^ T cells are not likely essential for regulating the immunopathology of schistosomiasis.

## Innate γδ T Cells in Schistosomiasis

The previously discussed T lymphocyte, the αβ T cell, has TCR composed of two glycoprotein chains, α and β, while γδ T cells, another subset of lymphocytes, are CD4^−^CD8^−^CD3^+^ T cells with TCR encoded by the γ and δ genes (122). Generally, γδ T cells consist of ~5% of the circulating peripheral blood T cells, but in some infectious diseases, the enumeration of γδ T cells exceeds 30% of the peripheral blood T cells (123). During schistosomiasis, the response to schistosome antigen is primarily mediated by activated CD4^+^ αβ T cells (33). However, it has also been reported that γδ T cells are recruited to egg-induced granuloma in the murine schistosomiasis (124) and that the levels of γδ T cells are increased in schitosome-infected mice and patients (125, 126). However, an earlier study investigating the relative roles of αβ and γδ T cells in the immunopathology of schistosomiasis found that mutant mice lacking γδ T cells display vigorous formation of egg granulomas similar to normal mice, which demonstrates that granuloma formation is not dependent on γδT cells (127). In contrast with this study, another study identified two different γδ T cell subsets, including the Vγ1 γδ T cells that secrete IFN-γ only and the Vγ2 γδ T cells that secrete both IL-17A and IFN-γ (74). During *S. japonicum* infection, Vγ2 γδ T cells prevent hepatic fibrosis by recruiting neutrophils and secreting IL-17A (74). Thus, the role of innate γδ T cells in the pathology of schistosomiasis requires further investigation.

## The Impact of Other Immune Cells on CD4^+^ T Cells and Regulation on Immunopathology of Schistosomiasis

Over the past four decades, immunoregulation has been deeply studied in the context of schistosomiasis. T-cell subsets are influenced by macrophages, B cells, DCs, and eosinophils, of which macrophages represent nearly 30% of the total granuloma cells. Macrophages, similar to Th1 and Th2 cells, can be classified into two major types, classically activated macrophages (CAM/M1) and alternatively activated macrophages (AAM/M2). CAM polarization, stimulated by inflammatory cytokines, such as IFN-γ, IL-12, and IL-18, serve a vital role in the response to intracellular pathogens like *Mycobacterium tuberculosis* (128), whereas AAM polarization is dependent on Th2-type cytokines, such as IL-4 and IL-13, that induce the expression of arginase-1 (Arg-1), Ym-1, and Fizzl, which is mainly involved in allergic, cellular, and humoral responses to parasites (129). Egg-induced inflammation and liver fibrosis promotes the Th2 response, which in turn increases AAM polarization in granulomas (130). In several models of Th1-polarized mice, such as IL-4/IL-10 deficient, IL-4/IL-13 deficient, and egg/IL-12-immunized or macrophage-specific IL-4 deficient (131, 132), schistosome infection failed to induce the expression of Arg-1, demonstrating that Th2 cytokines are necessary for the development of Arg-1-expressing AAM. Moreover, Th1-polarized mice showed an increased inducible nitric oxide synthase response together with smaller granulomas and elevated mortality (131, 132). Thus, CAM polarization is associated with Th1 responses and Arg-1-expressing AAM is associated with Th2 responses and liver fibrosis progression.

The function of B cells has also been intensely investigated in schistosomiasis. Several studies have demonstrated that the B-cell number in the lymph nodes and spleen significantly increase during the schistosome infection, suggesting that B cells are important for host responses against schistosome infection (133, 134). Mice immunized with radiation-attenuated cercariae of *S. mansoni* showed reduced protection against challenge infection in B-cell knockout mice compared to wild-type mice (135). IgE antibody against the worms, but not the eggs, has been closely associated with resistance to reinfection, and eosinophil-mediated antibody-dependent cellular cytotoxicity is the main immune mechanism to kill early schistosome larvae (136). Evidence shows that SEA-specific IgG1 is the main isotype released from the early granulomas and that, during the chronic infection stage, granulomas secrete a mixture of IgG1, IgG2, IgG3, and IgA (137). The antibodies produced within intragranulomas mainly function to downregulate the granuloma formation, and it has been found that immune complexes from chronic schistosomiasis patients can inhibit granuloma formation *in vitro* (138). In addition, research has been done on the regulatory role of B cells in schistosomiasis immunopathology and T-cell effector functions. A previous study has proven that B cells are important in promoting a strong Th2-type response to helminths (139). After SEA stimulation, mesenteric lymph cells from B-cell-deficient mice produce more Th1-type cytokines and less Th2-type cytokines compared to wild-type mice (140, 141). A recent study has shown that the absence of IL-4α signaling in B cells leads to increased mortality and pathology in *S. mansoni*-infected mice, which is attributed to decreased Th2 responses (142). These findings demonstrate that B cells are critical in directing and mediating Th cells toward Th2 responses. In addition, in a study about *S. mansoni*-mediated protection against experimental ovalbumin-induced allergic airway inflammation, the investigators found that splenic marginal zone CD1d^+^ B cells from schistosome-infected mice had the ability to secret IL-10 and induce generation of FoxP3^+^ Treg cells. Although B cells are essential for Th2 responses and Treg cell generation, B cells are not responsible for granuloma formation (140). However, a contrasting study showed that B-cell deficiency had no effect on Th2 responses but augmented tissue pathology (143). Therefore, further study is needed to clarify the role of B cells in immunopathology of schistosomiasis.

DCs are important antigen-presenting cells that have superior activity to stimulate naive T-cell activation and mediate the polarization of CD4^+^ T cells in response to invading pathogens. DCs determine Th differentiation through secreted polarizing cytokines. For example, *M. tuberculosis* infection induces DCs to produce IL-12 to elicit a strong IFN-γ-producing Th1 response, which leads to macrophage activation and killing of intracellular bacteria (144). Depletion of DCs in CD11c-diphtheria toxin receptor mice demonstrated that DCs are required to initiate Th2 responses in the *S. mansoni* infection (145). However, DC subsets, such as conventional CD11c^+^ DCs and some specialized DCs do not produce IL-4, which is important for Th2 polarization. Ma et al. uncovered a novel subset of CD11c^+^CD49b^+^FcεRI^+^ DCs that can produce IL-4 and subsequently promote Th2 differentiation in an IL-4-dependent manner (146). However, contradictory findings revealed that conventional DCs are critical subsets for Th2 effector cell development during acute *S. mansoni* infection (147). In fact, more researchers support the notion that DCs do not need to produce IL-4 to promote Th2 development (148–150) because IL-4-deficient DCs still show the ability to induce excellent Th2 responses (148). In addition, a recent study revealed an unrecognized role of type I IFN in the Th2 response, whereby IFN-I signaling is implicated in not only activating DCs but also enhancing DCs effective migration and antigen presentation during the *S. mansoni*-induced Th2 response (151). Thus, the exact mechanism by which DCs induce Th2 responses needs to be further clarified.

## Summary and Concluding Comments

**Figure 1:**
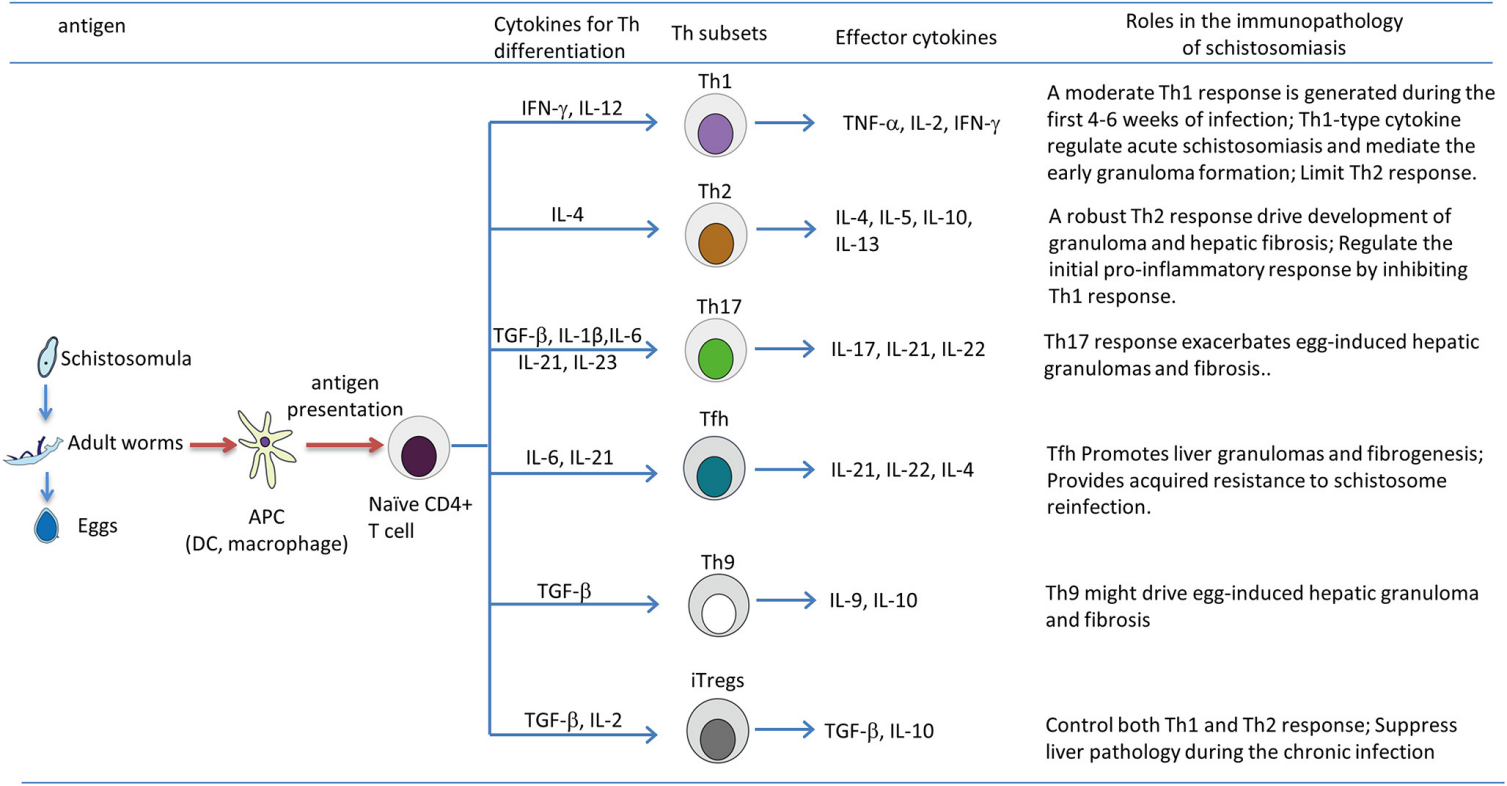
T helper (Th) subsets differentiation and their roles in immunopathology of the murine schistosomiasis. During the progression of schistosomiasis, the schistosome antigen from schistosomula, adult worms, or eggs are captured and processed by antigen-presenting cells, such as dendritic cells (DCs) and macrophages; this leads to the production of various cytokines, and processed antigens are presented to naive CD4^+^ T cell in the form of major histocompatibility complex (MHC) class II-peptide complexes, then activated CD4^+^ T cells differentiate into Th1, Th2, Th17, Tfh, Th9, and Tregs, which are strictly regulated by the local microenvironment (e.g., cytokines and antigens of schistosomes) and cross-talk with various immune cells (e.g., macrophages, B cells, and DCs) through a complicated interacting network. It is generally thought that Th2, Th17 responses and Tfh, Th9 cells positively drive the development of pathology of chronic schistosomiasis, whereas the Th1 response counteracts Th2-mediated chronic pathology, and Tregs exert a suppressive effect on the immunopathology of schistosomiasis by inhibiting both Th1 and Th2 responses.

Schistosomiasis is a disease with profound impact on human health. At least 230 million people worldwide are affected by the parasitic disease (1). In the near future, we may face a situation where there is no available drug to treat schistosomiasis, as praziquantel is still the only effective drug being used to treat the disease and praziquantel resistance has been reported in endemic areas and in the laboratory (152, 153). Therefore, it is urgent to develop some alternative drugs to control schistosomiasis, including the use of vaccines (154–156). Schistosome infection can lead to acute febrile illness and chronic life-threatening hepatosplenic disease. As summarized in Figure 1, during the progression of the disease, Th cells are activated and differentiated into distinct effector subsets, including Th1, Th2, Th17, Tfh, Th9, and Treg cells. Acute schistosomiasis is recognized as a Th1-dominated disease. Recent studies showed that Th17 and Tfh are also involved in the immune response of acute cases (71, 94). Chronic disease of hepatic granuloma formation and fibrosis are upregulated by Th2 and Th17 cells, mainly secreting IL-4 and IL-17, respectively (33, 63), and downregulated by Th1 and Treg cells (80, 113). The plasticity of Th cells are affected by the local microenvironment (e.g., cytokines and antigens of schistosomes) and regulated by various immune cells (e.g., macrophages, B cells, and DCs) through a complicated interacting network. Although we have acquired sufficient knowledge about the immunopathology of schistosomiasis, the effective strategies to restrain the development of granulomas and subsequent fibrosis are still lacking. A vaccine designed to skew the immune response toward the Th1 phenotype would be useful to prevent the development of fibrosis (80). However, murine studies have shown that extreme immune deviation toward either Th1 or Th2 results in increased pathology and premature death (18, 157). Most importantly, maintaining the balance of various effector T cells would be critical to prevent excessive pathology. While many researchers are focused on the Tregs-associated suppression and the powerful force of IL-10, successful immunotherapies will only be developed for schistosomiasis if we have a broader view and deeper understanding of the mechanism of T-cell-mediated liver immunopathology.

## Author Contributions

CL organized the article. BZ wrote the draft. JZ, HC, HN and HM participated in the conception, discussion and revision of the draft. QG edited the language and figure.

### Conflict of Interest

The authors declare that the research was conducted in the absence of any commercial or financial relationships that could be construed as a potential conflict of interest.
